# Supplementary material for: The causal relationship between the human gut microbiota and pyogenic arthritis: a Mendelian randomization study
Source: Front Cell Infect Microbiol. 2024 Nov 26;14:1452480. doi: 10.3389/fcimb.2024.1452480 (PMC11629706; doi:10.3389/fcimb.2024.1452480)
Supplement: Supplementary file 1 [file DataSheet1.zip › mendelian test/ebi-a-GCST90027586.csv_leave_one_out.pdf]

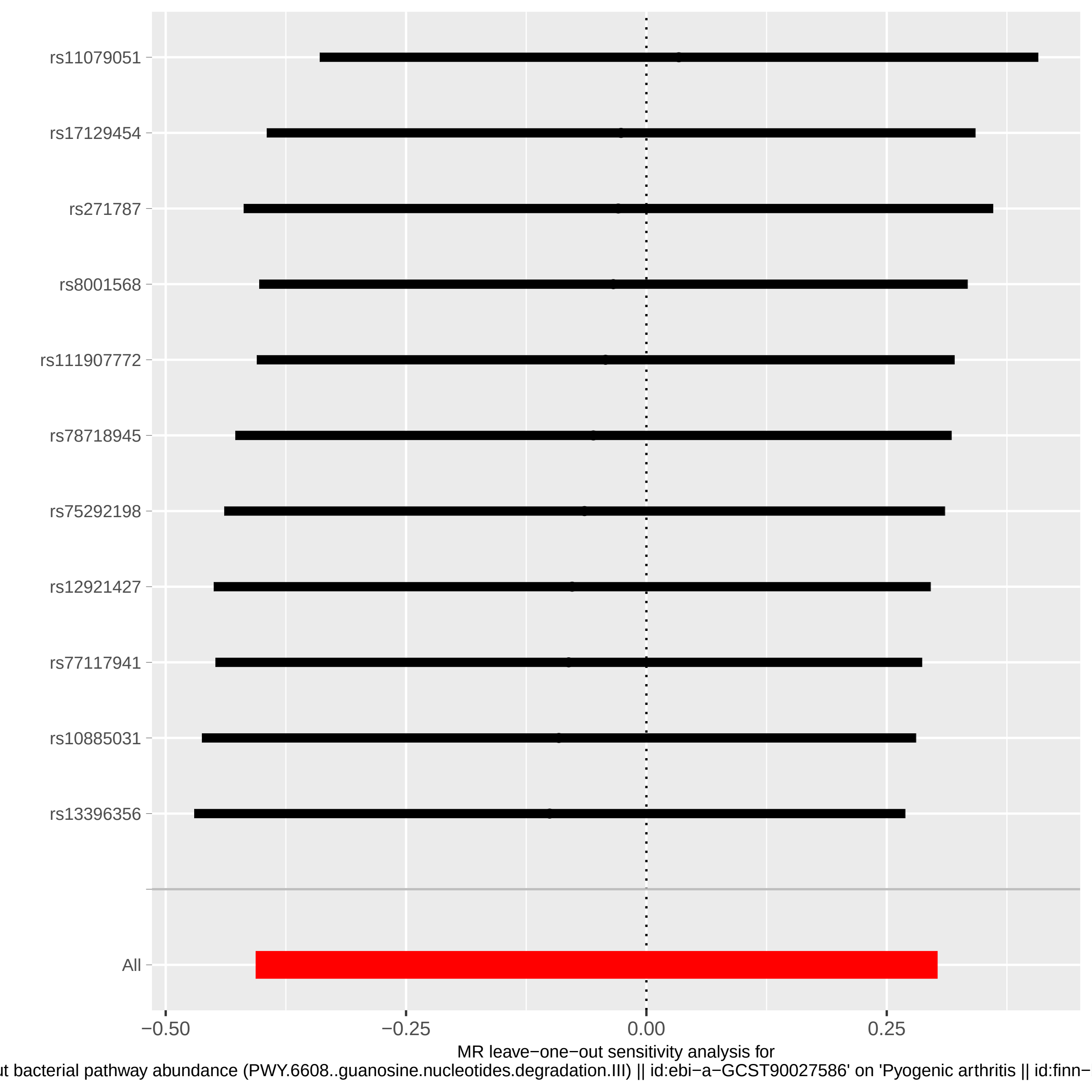

rs11079051
rs17129454
rs271787
rs8001568
rs111907772
rs78718945
rs75292198
rs12921427
rs77117941
rs10885031
rs13396356
All
−0.50
−0.25
0.00
0.25
MR leave−one−out sensitivity analysis for
ut bacterial pathway abundance (PWY.6608..guanosine.nucleotides.degradation.III) || id:ebi−a−GCST90027586' on 'Pyogenic arthritis || id:finn−
